# Supplementary material for: Association between oral microbiome and seven types of cancers in East Asian population: a two-sample Mendelian randomization analysis
Source: Front Mol Biosci. 2023 Nov 21;10:1327893. doi: 10.3389/fmolb.2023.1327893 (PMC10702768; doi:10.3389/fmolb.2023.1327893)
Supplement: Supplementary file 1 [file DataSheet1.ZIP › Supplementary Materials/MR plots of saliva/saliva═╝/Prostate cancer/pheno.3242_to_prostate cancer_forest.pdf]

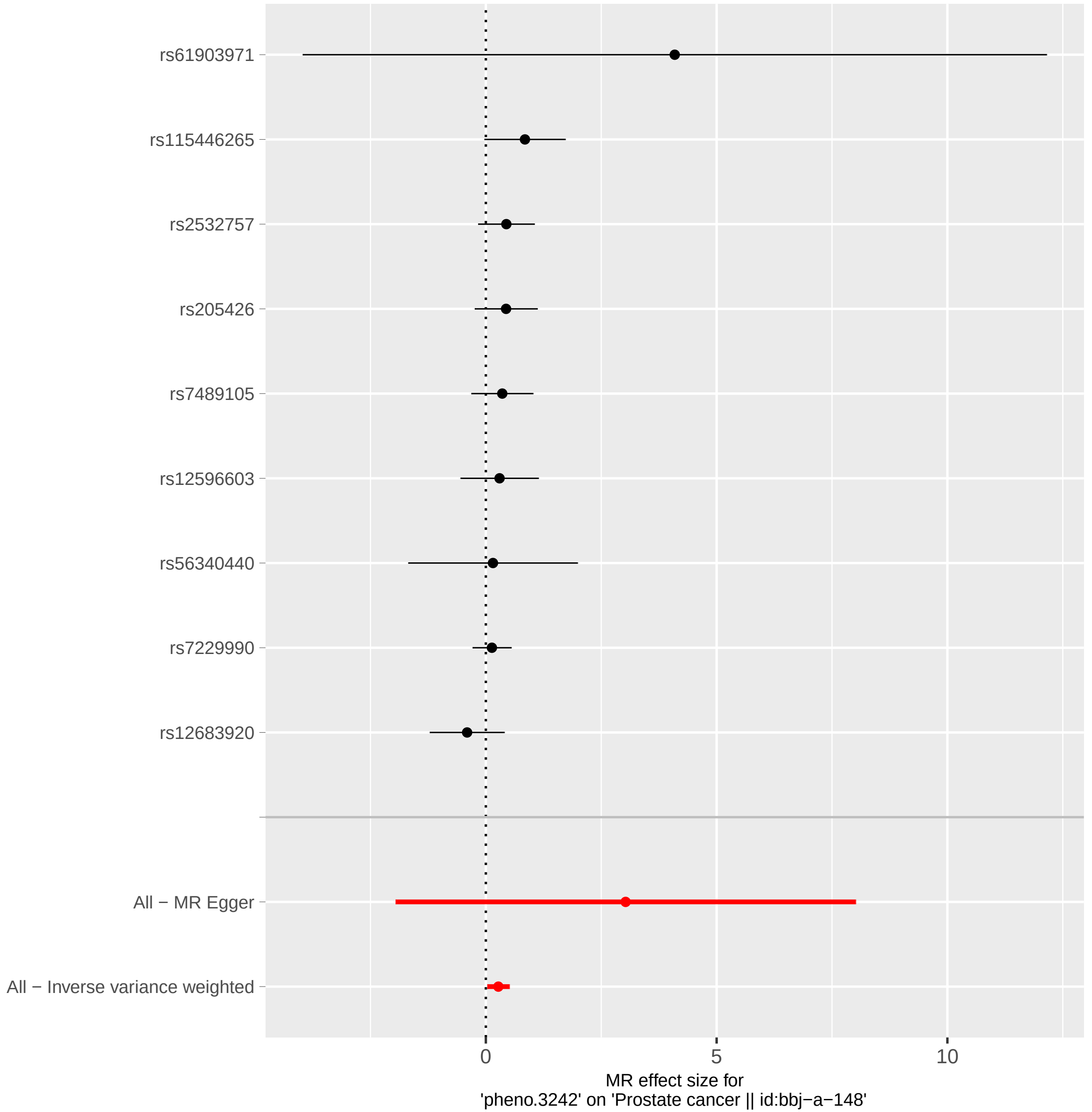
rs61903971
rs115446265
rs2532757
rs205426
rs7489105
rs12596603
rs56340440
rs7229990
rs12683920
All − MR Egger
All − Inverse variance weighted
0
5
10
MR effect size for
'pheno.3242' on 'Prostate cancer || id:bbj−a−148'
